# Do Exosomes Add Another Bi-Layer of Complexity to the Renin-Angiotensin System?

**DOI:** 10.34067/KID.0000000572

**Published:** 2024-11-21

**Authors:** Romer A. Gonzalez-Villalobos

**Affiliations:** Cardiopulmonary TA, Janssen Research and Development, LLC, Cambridge, MA

**Keywords:** hypertension, renin angiotensin system

Extracellular vesicles (EVs) are small, <5000 nm, spherical lipid membrane vesicles secreted into the extracellular space by numerous cell types. EVs contain proteins, nucleic acids, lipids, and other signaling cues and serve important roles in intercellular communication. Evidence suggests that EVs may play an important role in many cardiovascular and kidney diseases, including hypertension.^1^ Because their cargo reflects the physiological state of their cellular origin, numerous attempts have been made to develop methods to isolate EVs from fluids, including plasma and urine. The hope is to develop better biomarkers for diagnosis, patient stratification, target engagement, treatment response, *etc*. Besides this, the fact that EVs modify the behavior of target cells lends rationale to their potential as therapeutic tools.

In this issue of *Kidney360*, Ahmad *et al.* report the presence of renin-angiotensin system (RAS) components in EVs isolated from blood and urine of a small cohort of hypertensive patients.^2^ On the basis of their interest in the biology of Angiotensin (Ang)-(1–12), an emphasis was placed on determining the catalytic activity of angiotensin-generating enzymes, including angiotensin converting enzyme (ACE), ACE2, neprilysin, and chymase. Several thought-provoking findings are disclosed, including (*1*) ACE, ACE2, neprilysin, and chymase are present in blood and urine EVs, but their activity is higher in urine EVs; (*2*) chymase activity seems to be higher than other Ang-generating enzymes; and (*3*) there was small but statistically significant increase in the EV content of chymase when uncontrolled patients (BP ≥140/80 mm Hg) were compared with controlled individuals (BP <140/80 mm Hg).

As is often the case with any initial observations, we are left with more questions than answers. First, where do EVs come from? It is tempting to speculate that the endothelium and proximal tubule are likely sources of ACE-enriched EVs and that mast cells release chymase-enriched EVs. Yet, this needs to be confirmed. Second, how do EVs from hypertensive patients compare with EVs extracted from healthy individuals? Does hypertension cause an increased release of RAS-enriched EV, or does it change their enzymatic content? Previous studies show that EV abundance is increased by hypertension and hypertension-related injury^3–5^ and that there are specific EV signatures in patients affected by hypertension and kidney diseases.^1,6^ These questions contextualize the need for more research and point to EVs' potential to become part of the diagnostic and biomarker toolbox in hypertension, especially in the context of RAS alterations.

Fourth, and most importantly, what is the functional relevance of these findings? It is tempting to speculate that EVs may endow tissues with ACE and chymase-like activity to synthetize Ang peptides, including Ang-(1–12). It certainly has been demonstrated in preclinical species that EVs can transfer active RAS components.^7,8^ In the specific case of chymase, the authors point to recent findings by others and their observations to postulate an important role of chymase in human renal dysfunction and hypertension. Although this is enticing, there is no evidence from blind randomized placebo-control trials to support it. Indeed, results from recent clinical trials show that fulacimstat, a chymase inhibitor, was devoid of hemodynamic effects and failed to prevent adverse remodeling after myocardial infarction or to reduce albuminuria in patients with diabetic kidney disease.^9,10^

At the end, the most important contribution of Ahmad *et al.* is to bring light into the potential role of EV in regulating Ang peptide formation in humans. This is the sort of out-of-the box thinking that often paves the way for significant advancement. We should all be thankful to the authors for challenging our assumptions regarding the RAS.
